# Anti-HBs antibody status and some of its associated factors in dental health care workers in Tehran University of Medical Sciences: Anti-HBs Ab and associated factors in dental society

**Published:** 2011-02-01

**Authors:** Seyed Moayed Alavian, Nastaran Mahboobi, Nima Mahboobi

**Affiliations:** 1 Baqiyatallah Research center for Gastroenterology and Liver Diseases, Tehran, IR Iran; 2 Tehran Hepatitis Center, Tehran, IR Iran; 3 Department of Oral and Maxillofacial Surgery, Faculty of Dentistry, Tehran University of Medical Sciences, Tehran, IR Iran; 4 Students' Scientific Research Center, Tehran University of Medical sciences, Tehran, IR Iran

**Keywords:** Hepatitis B, Antibody, Vaccination, Dental health care providers

## Abstract

**Background:**

Dental health care providers are at risk of infection with hepatitis B virus (HBV). Dentists can occupationally become infected with HBV through needle sticks or exposure to blood and other body fluids.

**Objectives:**

To evaluate anti-HBs antibody titer in students, professors, clinical assistants and non-clinical staff of Faculty of Dentistry, Tehran University of Medical Sciences (TUMS), and to investigate the probable correlation between the level of immunity and a number of associated factors.

**Patients and Methods:**

230 participants who had a history of previous HBV vaccination (receiving at least two doses of HBV vaccine) and a negative history of being infected with HBV were studied. Participants' data were recorded using a checklist, and the level of antibody was measured by enzyme-linked immunosorbent assay (ELISA).

**Results:**

While there existed statistically significant correlations between age, occupation, smoking, complete and scheduled vaccination and time of the last vaccination with the level of anti-HBs antibody, the correlation between gender and level of the antibody was not significant. Multiple regression analysis revealed significant association between immune response and age and time of the last vaccination.

**Conclusions:**

Due to the significant correlation between younger age and anti-HBs antibody titer in our study, it makes sense to establish a mandatory complete and scheduled vaccination program for all members of dental society younger than 40 years.

## Background

By the beginning of the third millennium, hepatitis B is still a major public health problem globally. More than two billion people have been infected with this virus, and of these, more than 350 million suffer from chronic hepatitis B virus (HBV) infection worldwide [1]. Higher prevalence of HBV infection among dentists, especially dental surgeons, has been reported in serological studies from different parts of the world in comparison with general population [2][3][4]. Dentists can occupationally become infected with HBV through needle sticks or exposure to blood and other body fluids [3][5], and this might discourage them to work for infected patients [6]. So far, the most effective way which has been introduced to prevent HBV infection is vaccination [7]. As it is shown, not everybody responds to HBV vaccination and dentists should check their level of anti-HBs antibody titer when a complete scheduled vaccination program is accomplished [8][9]. Several factors such as age of >40 years, smoking, and obesity were reported to have contributed to inefficient immune response to HBV vaccine in non-respondents [7][10]. The rate of vaccination varies greatly and vaccination has not been accepted as a probable means of elimination of risk for occupational HBV infection by all dentists [11]. Despite remarkable scientific achievements during the last years, correlation between the titer of anti-HBs antibody and HBV infection risk has not been thoroughly clarified yet [2]. In Iran, it is estimated that over 35% of the general population have been exposed to HBV and approximately 2.5% are chronic carriers [12]. To the best of our knowledge, there is no documented data of infection rate in dentists and dental staff in Iran. In addition, no mandatory vaccination program against HBV for dentists exists in this country [13].

## Objectives

This study was conducted to determine anti-HBs antibody titer in students, professors and assistants of Faculty of Dentistry, Tehran University of Medical Sciences (TUMS) and to investigate the possible correlation between gender, age, smoking, occupation, complete and scheduled vaccination and time of the last vaccination with anti-HBs antibody titer in this population.

## Patients and Methods

In a cross-sectional study performed in October 2008, 230 students, professors, clinical assistants and non-clinical staff from Faculty of Dentistry, Tehran University of Medical Sciences (TUMS) who had a history of previous HBV vaccination (receiving at least two doses of HBV vaccine) and a negative history of being infected with HBV were studied. Written informed consent was taken from each participant. Five to 10 mL of venous blood was obtained from each participant at Faculty of Dentistry. Samples were sent to Valiasr laboratory, Imam Khomeini Hospital, affiliated to TUMS. The samples were centrifuged to separate the serum for antibody assay. The relative serum antibody titers were determined using an enzyme-linked immunosorbent assay (ELISA) (Diaplus kit through sandwiched noncompetitive ELISA). The titers were then classified into three groups as follows: 1) <10 IU/L which was considered "no or weak immunity". 2) 10-99 IU/L which was considered "relative immunity," and 3) > 100 IU/L which was considered "high immunity". A checklist was used to collect supplementary data including age, gender, occupation (i.e. student, professor, clinical assistants, or non-clinical staff), smoking status (i.e. smoker, non-smoker, or abandoned smoking), time of the last vaccination (which could be "< 5 years" or "≥ 5 years before") and receiving vaccination on schedule (which could be "yes" or "no"). X(2) was used to compare categorical variables. Multiple regression analysis was also used to evaluate correlations between variables. SPSS® ver 15 for Windows® (SPSS, Chicago, Ill., USA) was used for analysis. A p-value < 0.05 was considered statistically significant. Participants with anti-HBs antibody titers < 10 IU/L, received a booster dose.

## Results

Two-hundred and thirty participants including109 (47.4%) students, 13 (5.7%) professors, 61 (26.5%) clinical dental assistants and 47 (20.4) non-clinical staff were studied. There were 75 (32.6%) men and 155 (67.4%) women. Thirty (13.0%) participants were not immune; 41 (17.8%) were relatively immune, and 159 (69.1%) were immune. No significant relation was found between gender and anti-HBs antibody titer (p = 0.481). Relation between age and immunization status is summarized in Table 1. A statistically significant correlation was found between age and level of immunity (p = 0.021) (Table 1). Of 230 studied participants, 165 were non-smokers, 47 were smokers and 16 had quitted smoking. There was a significant (p < 0.001) correlation between smoking and serum anti-HBs titer. One-hundred and eighty (78.3%) participants mentioned that they received vaccination less than five years before, while others (50 participants; 21.7%) stated that they were vaccinated more than five years before. A statistically significant correlation was found between time of the last vaccination and anti-HBs antibody titer. Participants with longer interval (= 5 years) had lower immune response to the vaccines (p < 0.001). Only 122 participants (53.0%) had received a complete course of vaccination. A significant correlation (p = 0.008) was found between the immunity level and completing a correct schedule of vaccination (Table 1). Furthermore, 50.7% of men (n = 38) and 54.2% of women (n = 81) had received their vaccines completely and well-scheduled. No statistically significant correlation was found between gender and complete and well-scheduled vaccination (p = 0.252). Of 180 participants, who had been vaccinated < 5 years before, 107 (59.4%) had received it on schedule. This was significantly different (p < 0.001) from that in another study group-only 15 (30.0%) participants of those who had received vaccine = 5 years before reported that they had received a complete and on schedule vaccination. The correlation of immunity status with occupation is shown in Table 1. A statistically significant different level of immunity was found between the four studied career groups. In multiple regression analysis, only age and time of the last vaccination were found to be independent predictors of immune system response (p < 0.001 for both variables).

**Table 1:** Relationship between age and immunization status

**Variable**	**Non-immune**	**Relatively immune**	**Immune**	**p-value**
**Age groups**				0.021
**20–29** (n = 104)	4.8% (n = 5)	18.2% (n = 19)	77% (n = 80)	
**30–39** (n = 54)	18.5% (n = 10)	20.4% (n = 11)	61.1% (n = 33)	
**40–49** (n = 65)	23.1% (n = 15)	16.9% (n = 11)	60.0% (n = 39)	
**50–59** (n = 7)	0.0% (n = 0)	0.0% (n = 0)	100% (n = 7)	
**Vaccination on schedule**				0.008
**Yes** (n = 122)	6.6% (n = 8)	18.8% (n = 23)	74.6% (n = 91)	
**No** (n = 108)	20.4% (n = 22)	16.7% (n = 18)	63% (n = 68)	
**Occupation**				0.005
**Dental students **(n = 109)	6.4% (n = 7)	17.4% (n = 19)	76.2% (n = 83)	
**Professor**s (n = 13)	0 (n = 0)	23.1% (n = 3)	76.9% (n = 10)	
**Clinical ssistants** (n = 61)	14.8% (n = 9)	18% (n = 11)	67.2% (n = 41)	
**Non-clinical staff **(n = 47)	29.8% (n = 14)	17.0% (n = 8)	53.2% (n = 25)	

## Discussion

The objective of this study was to assess anti-HBs antibody titer in vaccinated dental students, professors, clinical assistants and non-clinical staff and to assess its correlation with gender, age, occupation, smoking status and time of the last vaccination in Faculty of Dentistry, TUMS. Our results indicated that while a statistically significant correlation was found between antibody titer and age, occupation, smoking, complete scheduled vaccination and time of the last vaccination; no significant correlation was observed between gender and anti-HBs antibody titer. In our survey only those who had received at least two doses of HBV vaccine were included. The rate of immunity was almost 70% while approximately 18% were relatively immune and others were non-immune. A similar study on general dentists in Iran showed that 77% of the participants were completely immune, while 17% were relatively immune and 6% were non-immune [2]. McCarthy and Britton in a questionnaire-based study on final year dental students of The University of Western Ontario in Canada showed that 6% of dental students' response to vaccination against HBV was inadequate [14]. Another study from South Korea by Song and colleagues [15] revealed that 49.7% of dentists, 25% of hygienists, and 32.6% of assistants knew that they were anti-HBs antibody positive while others mentioned that they were not immune or they did not know about their antibody level. In this study, only 53% of participants mentioned that they had received vaccine on time, which is in contrast to the results of Moore, et al, who demonstrated that 96.7% of their study population (i.e., dentists, nurses, hygienists, technicians, etc) received complete vaccination [16]. This variation might be attributed to high number of students in our study sample who were receiving their vaccine on schedule but they were in the middle of their vaccination program. In another study from Guilan province, northern Iran, Rabi'ei, et al, showed that 71.9% of their population received complete vaccination which was 100% of professional dentists, 94.9% of general dentists and 50% of dental assistants [17]. In a study on general dentists in Iran, Alavian, et al, illustrated that 82.1% of the target population received all doses of vaccination on schedule [2]. In a study in UK, 97% of dentists stated that they had been immunized against HBV [18]. In a study by Song in South Korea, 63.3% of dentists, 62.7% of hygienists, and 65.2% of assistants (a total of 63.3% of the participants) mentioned that they received vaccination [15]. Batista, et al, in a study in Brazil reported that 73.1% of their vaccinated participants received all three recommended doses of vaccination against HBV [10]. In a study on dentists in Romania, 67% of participants reported that they had not been vaccinated, 4% had received two doses, and only 26% had received all three recommended doses of vaccine [19]. A significant correlation was found between lower age and level of immunity in our study which confirmed the results of Batista, et al [10] and Alavian, et al [2]. These findings suggest that the serum level of antibody may decrease with increasing age. Therefore, it seems necessary for dental health care workers to be immunized before the age of 40 years. Our findings showed a statistically significant correlation between smoking and decreased immune response to HBV vaccine. Nonetheless, this finding was not confirmed by multiple regression analysis that is in line with the results of Alavian and colleagues [2]. It can be inferred that to enhance immane system response, dental health care workers, as members of a society which is at high risk of HBV infection, should not smoke. In our study, participants with longer interval between their last vaccination date and time of the present study revealed lower level of immunity in comparison with those whose vaccination was completed less than five years before. Alavian and colleagues also showed a significant correlation between receiving vaccine less than eight years prior to their study and higher immunity level [2]. On the other hand, more participants with shorter interval between completion of their vaccination and this study had received HBV vaccine completely and on schedule. Therefore, it can be construed that both of these characteristics (vaccination more than five years and an incomplete vaccination) have negative influence on the immune response. In our study, the immune response was significantly higher among dental students and professors in comparison with clinical assistants and non-clinical staff. It can be inferred that this characteristic is a result of higher emphasis on importance of vaccination in dental students' educational curriculum. Unfortunately, although a free vaccination against HBV is available in health care offices in Iran, no mandatory vaccination program against HBV has been established for dentists yet. The sorrowful penalty of those who were not immune against HBV and have been infected is so terrible that policy makers should establish a mandatory vaccination program in dental schools as soon as possible. Since in this study direct method was implemented to evaluate the serum level of anti-HBs antibody in the target population, it can be claimed that more accurate results have been achieved in comparison with many of previous studies. Furthermore, studying of various groups of dental society can be considered as another strength point of this study. One of the limitations of our study was that our data were collected from volunteer participants of Faculty of Dentistry, TUMS, hence, our findings may not represent the whole population of Iranian dentists. In addition, questionnaire-based data gathering often suffers from recall bias. The fact that a small number of professors participated in the present study may limit the external validity of the results. In conclusion, the results of the present study showed that there is a significant correlation between level of anti-HBS antibody and age, occupation, smoking, complete scheduled vaccination and time of the last vaccination. The results of multiple regression analysis showed direct association between higher age and time of the last vaccination and lower immune response. Due to a significant correlation which has been found between the younger age and immune response to HBV vaccine, a mandatory vaccination program for all members of dental society before the age of 40 makes sense. Furthermore, infection control educational courses will be most helpful for clinical assistants and non-clinical staff to better understand the characteristic of HBV and the importance of their anti-HBs antibody titer. To investigate more contributory factors in immune response to vaccination against HBV in dental society and in other health care workers, more studies are suggested.
